# Supplementary material for: Modeling glioblastoma heterogeneity as a dynamic network of cell states
Source: Mol Syst Biol. 2021 Sep 16;17(9):e10105. doi: 10.15252/msb.202010105 (PMC8444284; doi:10.15252/msb.202010105)
Supplement: Supplementary file 6 — Source Data for Figure 5 [file MSB-17-e10105-s004.zip › Figure5A_sourcedata/GSEA_3071/nosigdb_stateIII.GseaPreranked.1622795155562/RICHARDS_NOWAKOWSKI_SCIENCE_2017_IPC_NEN3_UPREG.html]

Details for gene set RICHARDS\_NOWAKOWSKI\_SCIENCE\_2017\_IPC\_NEN3\_UPREG[GSEA]

|  || Dataset | state33071 |
| Phenotype | NoPhenotypeAvailable |
| Upregulated in class | na\_pos |
| GeneSet | RICHARDS\_NOWAKOWSKI\_SCIENCE\_2017\_IPC\_NEN3\_UPREG |
| Enrichment Score (ES) | 0.24648438 |
| Normalized Enrichment Score (NES) | 3.4694326 |
| Nominal p-value | 0.0 |
| FDR q-value | 0.0 |
| FWER p-Value | 0.0 |
Table: GSEA Results Summary

  

Fig 1: Enrichment plot: RICHARDS\_NOWAKOWSKI\_SCIENCE\_2017\_IPC\_NEN3\_UPREG      
 Profile of the Running ES Score & Positions of GeneSet Members on the Rank Ordered List

  

| PROBE | GENE SYMBOL | GENE\_TITLE | RANK IN GENE LIST | RANK METRIC SCORE | RUNNING ES | CORE ENRICHMENT || 1 | RPLP0 |  |  | 6 | 0.633 | 0.0105 | Yes |
| 2 | RPL12 |  |  | 12 | 0.579 | 0.0202 | Yes |
| 3 | RPS25 |  |  | 19 | 0.527 | 0.0286 | Yes |
| 4 | RPLP1 |  |  | 25 | 0.514 | 0.0371 | Yes |
| 5 | RPL28 |  |  | 32 | 0.498 | 0.0450 | Yes |
| 6 | RPLP2 |  |  | 36 | 0.489 | 0.0535 | Yes |
| 7 | POLR2L |  |  | 38 | 0.482 | 0.0624 | Yes |
| 8 | RPS6 |  |  | 41 | 0.480 | 0.0711 | Yes |
| 9 | RPL8 |  |  | 44 | 0.469 | 0.0795 | Yes |
| 10 | RPL13A |  |  | 45 | 0.467 | 0.0883 | Yes |
| 11 | RPS5 |  |  | 57 | 0.454 | 0.0941 | Yes |
| 12 | RPS18 |  |  | 61 | 0.451 | 0.1019 | Yes |
| 13 | RPL36 |  |  | 62 | 0.449 | 0.1105 | Yes |
| 14 | RPS24 |  |  | 69 | 0.444 | 0.1173 | Yes |
| 15 | PDCD5 |  |  | 77 | 0.435 | 0.1238 | Yes |
| 16 | RPS19 |  |  | 84 | 0.427 | 0.1303 | Yes |
| 17 | NPM1 |  |  | 88 | 0.421 | 0.1376 | Yes |
| 18 | RPS27A |  |  | 95 | 0.413 | 0.1439 | Yes |
| 19 | RPL35A |  |  | 96 | 0.413 | 0.1517 | Yes |
| 20 | RPSA |  |  | 108 | 0.405 | 0.1566 | Yes |
| 21 | RPS27 |  |  | 132 | 0.388 | 0.1579 | Yes |
| 22 | DNAJC8 |  |  | 136 | 0.384 | 0.1644 | Yes |
| 23 | RPL21 |  |  | 141 | 0.382 | 0.1706 | Yes |
| 24 | RPL13 |  |  | 152 | 0.372 | 0.1751 | Yes |
| 25 | GLO1 |  |  | 161 | 0.367 | 0.1800 | Yes |
| 26 | COMMD6 |  |  | 173 | 0.359 | 0.1839 | Yes |
| 27 | RPL18 |  |  | 178 | 0.355 | 0.1896 | Yes |
| 28 | RPS15 |  |  | 180 | 0.353 | 0.1961 | Yes |
| 29 | RPS27L |  |  | 188 | 0.348 | 0.2009 | Yes |
| 30 | GSTP1 |  |  | 211 | 0.333 | 0.2014 | Yes |
| 31 | PRDX1 |  |  | 220 | 0.328 | 0.2056 | Yes |
| 32 | METAP2 |  |  | 236 | 0.318 | 0.2077 | Yes |
| 33 | RPS8 |  |  | 248 | 0.311 | 0.2107 | Yes |
| 34 | RPL23 |  |  | 250 | 0.310 | 0.2164 | Yes |
| 35 | RPS16 |  |  | 256 | 0.308 | 0.2209 | Yes |
| 36 | C8orf59 |  |  | 263 | 0.304 | 0.2251 | Yes |
| 37 | RPL41 |  |  | 265 | 0.303 | 0.2306 | Yes |
| 38 | HNRNPA1 |  |  | 270 | 0.302 | 0.2354 | Yes |
| 39 | TMSB4X |  |  | 273 | 0.300 | 0.2405 | Yes |
| 40 | RPL10 |  |  | 285 | 0.296 | 0.2433 | Yes |
| 41 | TMEM258 |  |  | 295 | 0.292 | 0.2465 | Yes |
| 42 | BTF3 |  |  | 344 | 0.279 | 0.2392 | No |
| 43 | ZC3H15 |  |  | 347 | 0.278 | 0.2440 | No |
| 44 | CTTN |  |  | 406 | 0.258 | 0.2337 | No |
| 45 | ELAVL2 |  |  | 695 | -0.264 | 0.1631 | No |
| 46 | GPC2 |  |  | 717 | -0.265 | 0.1626 | No |
| 47 | FRMD4A |  |  | 819 | -0.270 | 0.1413 | No |
| 48 | LZTS1 |  |  | 837 | -0.271 | 0.1420 | No |
| 49 | HES6 |  |  | 860 | -0.272 | 0.1414 | No |
| 50 | LIMD1 |  |  | 962 | -0.277 | 0.1201 | No |
| 51 | SCHIP1 |  |  | 995 | -0.279 | 0.1171 | No |
| 52 | ZNF232 |  |  | 1086 | -0.284 | 0.0988 | No |
| 53 | SLC39A11 |  |  | 1182 | -0.289 | 0.0794 | No |
| 54 | TMEM108 |  |  | 1200 | -0.290 | 0.0805 | No |
| 55 | SLC4A7 |  |  | 1314 | -0.295 | 0.0564 | No |
| 56 | BTG2 |  |  | 1350 | -0.297 | 0.0529 | No |
| 57 | CCDC88C |  |  | 1378 | -0.299 | 0.0515 | No |
| 58 | LAPTM4A |  |  | 1391 | -0.299 | 0.0541 | No |
| 59 | CDON |  |  | 1423 | -0.301 | 0.0517 | No |
| 60 | ZEB1 |  |  | 1569 | -0.310 | 0.0195 | No |
| 61 | MED12L |  |  | 1593 | -0.311 | 0.0194 | No |
| 62 | ZC3H12C |  |  | 1673 | -0.317 | 0.0047 | No |
| 63 | MEX3A |  |  | 1749 | -0.322 | -0.0089 | No |
| 64 | NFIX |  |  | 1848 | -0.327 | -0.0284 | No |
| 65 | DAAM1 |  |  | 1915 | -0.330 | -0.0394 | No |
| 66 | SALL1 |  |  | 1962 | -0.333 | -0.0451 | No |
| 67 | ZNF292 |  |  | 1992 | -0.335 | -0.0464 | No |
| 68 | RASA4 |  |  | 2013 | -0.336 | -0.0452 | No |
| 69 | GTF2I |  |  | 2067 | -0.339 | -0.0527 | No |
| 70 | MFAP2 |  |  | 2169 | -0.347 | -0.0726 | No |
| 71 | KIF26B |  |  | 2291 | -0.356 | -0.0975 | No |
| 72 | MAGI1 |  |  | 2340 | -0.361 | -0.1033 | No |
| 73 | IRGQ |  |  | 2428 | -0.368 | -0.1191 | No |
| 74 | ZKSCAN1 |  |  | 2437 | -0.368 | -0.1142 | No |
| 75 | TARS2 |  |  | 2449 | -0.369 | -0.1100 | No |
| 76 | CLASRP |  |  | 2451 | -0.369 | -0.1033 | No |
| 77 | DENND1B |  |  | 2524 | -0.375 | -0.1150 | No |
| 78 | SDC2 |  |  | 2537 | -0.376 | -0.1110 | No |
| 79 | BAZ2B |  |  | 2576 | -0.380 | -0.1138 | No |
| 80 | TMTC2 |  |  | 2616 | -0.383 | -0.1167 | No |
| 81 | PAX6 |  |  | 2619 | -0.384 | -0.1099 | No |
| 82 | PLCB4 |  |  | 2735 | -0.394 | -0.1326 | No |
| 83 | SLC22A23 |  |  | 2842 | -0.402 | -0.1528 | No |
| 84 | SVIL |  |  | 2912 | -0.409 | -0.1631 | No |
| 85 | AASS |  |  | 2929 | -0.412 | -0.1595 | No |
| 86 | EGR1 |  |  | 2961 | -0.415 | -0.1597 | No |
| 87 | CCND2 |  |  | 3037 | -0.425 | -0.1713 | No |
| 88 | PTPRS |  |  | 3062 | -0.427 | -0.1695 | No |
| 89 | GPRC5B |  |  | 3092 | -0.430 | -0.1689 | No |
| 90 | ENC1 |  |  | 3169 | -0.440 | -0.1805 | No |
| 91 | MYO10 |  |  | 3196 | -0.442 | -0.1789 | No |
| 92 | MSMO1 |  |  | 3241 | -0.450 | -0.1819 | No |
| 93 | CD63 |  |  | 3258 | -0.452 | -0.1775 | No |
| 94 | ZNF253 |  |  | 3333 | -0.467 | -0.1880 | No |
| 95 | CELSR2 |  |  | 3378 | -0.475 | -0.1905 | No |
| 96 | AKNA |  |  | 3388 | -0.479 | -0.1837 | No |
| 97 | NEDD4L |  |  | 3406 | -0.481 | -0.1790 | No |
| 98 | FOXP2 |  |  | 3453 | -0.494 | -0.1817 | No |
| 99 | SLC1A3 |  |  | 3516 | -0.513 | -0.1882 | No |
| 100 | SYNE2 |  |  | 3523 | -0.518 | -0.1799 | No |
| 101 | ZFHX4 |  |  | 3540 | -0.524 | -0.1742 | No |
| 102 | RPN2 |  |  | 3581 | -0.539 | -0.1744 | No |
| 103 | DACH1 |  |  | 3607 | -0.553 | -0.1704 | No |
| 104 | RGS16 |  |  | 3616 | -0.555 | -0.1620 | No |
| 105 | CHD7 |  |  | 3622 | -0.561 | -0.1526 | No |
| 106 | SIPA1L2 |  |  | 3650 | -0.579 | -0.1487 | No |
| 107 | RFTN2 |  |  | 3654 | -0.582 | -0.1384 | No |
| 108 | MLC1 |  |  | 3659 | -0.584 | -0.1283 | No |
| 109 | FGD4 |  |  | 3692 | -0.610 | -0.1251 | No |
| 110 | CA12 |  |  | 3694 | -0.611 | -0.1137 | No |
| 111 | MPPED2 |  |  | 3736 | -0.655 | -0.1120 | No |
| 112 | INSM1 |  |  | 3772 | -0.704 | -0.1078 | No |
| 113 | COL11A1 |  |  | 3828 | -0.788 | -0.1072 | No |
| 114 | NKAIN3 |  |  | 3836 | -0.803 | -0.0938 | No |
| 115 | FJX1 |  |  | 3843 | -0.821 | -0.0798 | No |
| 116 | DLL3 |  |  | 3873 | -0.948 | -0.0693 | No |
| 117 | POU3F2 |  |  | 3875 | -0.953 | -0.0514 | No |
| 118 | C1orf61 |  |  | 3879 | -0.986 | -0.0334 | No |
| 119 | MFAP4 |  |  | 3897 | -1.102 | -0.0169 | No |
| 120 | NFIA |  |  | 3910 | -1.317 | 0.0050 | No |
Table: GSEA details [plain text format]

  

Fig 2: RICHARDS\_NOWAKOWSKI\_SCIENCE\_2017\_IPC\_NEN3\_UPREG: Random ES distribution      
 Gene set null distribution of ES for **RICHARDS\_NOWAKOWSKI\_SCIENCE\_2017\_IPC\_NEN3\_UPREG**
